# Non-canonical bases differentially represented in the sex chromosomes of the dioecious plant *Silene latifolia*

**DOI:** 10.1093/jxb/erae178

**Published:** 2024-04-23

**Authors:** Marcel Hubinský, Roman Hobza, Marta Starczak, Daniel Gackowski, Zdeněk Kubát, Tomáš Janíček, Lucie Horáková, Jose Luis Rodriguez Lorenzo

**Affiliations:** Department of Plant Developmental Genetics, Institute of Biophysics of the Czech Academy of Sciences, Královopolská 135, 612 65 Brno, Czech Republic; National Centre for Biomolecular Research, Faculty of Science, Masaryk University, Kamenice 5, 625 00 Brno, Czech Republic; Department of Plant Developmental Genetics, Institute of Biophysics of the Czech Academy of Sciences, Královopolská 135, 612 65 Brno, Czech Republic; Department of Clinical Biochemistry, Faculty of Pharmacy, Ludwik Rydygier Collegium Medicum in Bydgoszcz, Nicolaus Copernicus University in Toruń, ul. Karlowicza 24, PO-85-092, Bydgoszcz, Poland; Department of Clinical Biochemistry, Faculty of Pharmacy, Ludwik Rydygier Collegium Medicum in Bydgoszcz, Nicolaus Copernicus University in Toruń, ul. Karlowicza 24, PO-85-092, Bydgoszcz, Poland; Department of Plant Developmental Genetics, Institute of Biophysics of the Czech Academy of Sciences, Královopolská 135, 612 65 Brno, Czech Republic; Department of Plant Developmental Genetics, Institute of Biophysics of the Czech Academy of Sciences, Královopolská 135, 612 65 Brno, Czech Republic; Department of Plant Developmental Genetics, Institute of Biophysics of the Czech Academy of Sciences, Královopolská 135, 612 65 Brno, Czech Republic; Department of Plant Developmental Genetics, Institute of Biophysics of the Czech Academy of Sciences, Královopolská 135, 612 65 Brno, Czech Republic; MPI of Molecular Plant Physiology, Germany

**Keywords:** Cytosine modifications, dosage compensation, oxi-mCs, sex chromosomes, *Silene latifolia*, transposable elements

## Abstract

The oxidation of 5-methylcytosine (5mC) to 5-hydroxymethylcytosine (5hmC), 5-formylcytosine (5fC), and 5-carboxylcytosine (5caC), known as oxi-mCs, garners significant interest in plants as potential epigenetic marks. While research in mammals has established a role in cell reprogramming, carcinogenesis, and gene regulation, their functions in plants remain unclear. In rice, 5hmC has been associated with transposable elements (TEs) and heterochromatin. This study utilizes *Silene latifolia*, a dioecious plant with heteromorphic sex chromosomes and a genome with a large proportion of TEs, which provides a favourable environment for the study of oxi-mCs in individual sexes. Notably, we detected surprisingly high levels of oxi-mCs in *S. latifolia* comparable with mammals. Nuclei showed enrichment in heterochromatic regions, except for 5hmC whose signal was homogeneously distributed. Intriguingly, the same X chromosome in females displayed overall enrichment of 5hmC and 5fC compared with its counterpart. This fact is shared with 5mC, resembling dosage compensation. Co-localization showed higher correlation between 5mC and 5fC than with 5hmC, indicating no potential relationship between 5hmC and 5fC. Additionally, the promoter of several sex-linked genes and sex-biased TEs clustered in a clear sex-dependent way. Together, these findings unveil a hypothetical role for oxi-mCs in *S. latifolia* sex chromosome development, warranting further exploration.

## Teaser

This work reveals a differential distribution of oxidized methylcytosines in *Silene latifolia* sex chromosomes, and differential sex-specific clustering of selected sex-biased transposable elements and genes based on these modifications.

## Introduction

DNA methylation at the C-5 position of a cytosine residue represents one of the key processes involved in transposon silencing, development, and transcriptional control of gene expression (Slotkin and Martienssen, 2007; Pikaard and Mittelsten Scheid, 2014; Zhang *et al.*, 2018). In plants, methylated cytosine (5-methylcytosine, 5mC) occurs in all sequence contexts CG, CHG, and CHH (where H represents A, T, or C) (Cao and Jacobsen, 2002) and is recognized as the fifth nucleotide base. Plants possess mechanisms for cytosine demethylation. Passive demethylation occurs through reduced or inactive DNA methylation enzymes during replication, which are reduced or inactivated. Meanwhile, active demethylation happens through DNA glycosylase enzymes followed by base excision repair, which then replaces the methylated cytosine with an unmethylated residue (Li *et al.*, 2018). In mammals, three additional intermediates of active demethylation catalysed by the TET (ten–eleven translocation) family of Fe^2+^- and 2-oxoglutarate-dependent enzymes were discovered. These enzymes convert 5mC to oxidized derivatives (oxi-mCs), namely 5-hydroxymethylcytosine (5hmC), 5-formylcytosine (5fC), and 5-carboxycytosine (5caC) (Tahiliani *et al.*, 2009; He *et al.*, 2011; Pfaffeneder *et al.*, 2011). TET homologues were also confirmed in algae, metazoans, and fungi (Moroz *et al.*, 2014; Zhang *et al.*, 2014; Aravind *et al.*, 2019). It is known that oxi-mCs are not only intermediates of active demethylation, but are also present as stable epigenetic marks (Kriaucionis and Heintz, 2009; Bachman *et al.*, 2015). They are involved in important biological processes ranging from stem cell pluripotency, epigenetic reprogramming, and gene expression to neuron maturation (Wossidlo *et al.*, 2011; Wu *et al.*, 2011; Zhang and Zhou, 2019; Stoyanova *et al.*, 2021). In plants, TET homologues are unidentified but, TET-mediated epimutagenesis of the *Arabidopsis thaliana* methylome demonstrated enzymatic machinery able to reduce 5mC from DNA (Ji *et al.*, 2018). Previous studies in plants have led to conflicting conclusions, on the one hand with claims of low levels of 5hmC in *A. thaliana* (Erdmann *et al.*, 2015), and on the other hand demonstrating the presence of all oxi-mCs in different plant species (Tang *et al.*, 2014). Previous research on conifers, which are known to possess large numbers of repetitive sequences, indicates a functional role for cytosine modifications (Yakovlev *et al.*, 2019). It has been shown that oxi-mCs are enriched in the sequence of different transposable element (TE) families and potentially can regulate their activity in mammals and fungi in which they may be important components of centromeres (Chavez *et al.*, 2014; Deniz *et al.*, 2019). Research in rice led to similar findings, suggesting a possible role in the regulation of TEs by 5-hmC (Wang *et al.*, 2015). TEs, comprising large portions of plant genomes, exhibit significant variation in copy number and distribution across species (Kejnovsky *et al.*, 2012). Repetitive DNA accumulation has an important role in sex chromosome evolution and epigenetics (Kejnovsky *et al.*, 2009; Hobza *et al.*, 2015, 2017). In melon, a plant without sex chromsomes but with sex determination genes, an insertion of the hAT DNA transposon triggers the methylation of the transcription factor CMWIP1, leading to gynoecy (Martin *et al.*, 2009).

*Silene latifolia* is a dioecious plant with cytologically well distinguishable heteromorphic sex chromosomes (Kejnovsky and Vyskot, 2010). In the X:Y system present in *S. latifolia*, the lack of recombination in the sex-specific region of the Y chromosome leads to an accumulation of deleterious mutations and degeneration originating two different evolutionary strata (Bergero *et al.*, 2007). However, not only gene degeneration but also DNA rearrangements and the clustering of satellites, microsatellites, plastid sequences, and transposons in sex chromosomes is broadly documented, and specific repetitive sequences can also be differentially found between X and Y chromosomes (Hobza *et al.*, 2006; Cermak *et al.*, 2008; Puterova *et al.*, 2018). This abundance of TEs in plants with heteromorphic sex chromosomes presents a compelling opportunity to investigate the presence and distribution of oxi-mCs in individual sexes (Hobza *et al.*, 2017). The present study is aimed to profile the global levels of three oxi-mCs in both sexes of *S. latifolia,* visualize their distribution and describe the global pattern in interphase nuclei and metaphase chromosomes, and finally to investigate a potential relationship of sex-biased genes and TE clusters with oxi-mCs.

## Materials and methods

### Plant material

This study employed a well-established inbred population (U17, 17 generations of full-sib mating) of both male and female *S. latifolia* specimens obtained from the Institute of Biophysics collection in Brno, Czech Republic. The plants were cultivated under controlled greenhouse conditions under 50–60 μmol photons m^–2^ s^–1^ provided by an array of fluorescent tubes, following a standard long-day photoperiod (22 °C, 16 h light/8 h dark). For DNA immunoprecipitation and subsequent two-dimensional ultra-performance LC with tandem MS and UV detection (2D-UPLC-MS/MS) analysis, genomic DNA was isolated from young leaves in a vegetative state (~1-month-old) using a rapid nitrogen flash-freezing technique. Metaphase chromosomes and interphase nuclei, required for immunostaining, were obtained from synchronized root tips following the protocol outlined by Lengerova *et al.* (2004).

### DNA immunoprecipitation

DNA was extracted using a NucleoSpin® Plant II DNA Kit (Macherey Nagel, Düren, Germany) according to the manufacturer’s instructions. The DNA immunoprecipitation (DIP) assay was performed according to Rodríguez Lorenzo *et al.* (2020). Antibodies used in DIP were anti 5-mC (Abcam, cat. no. ab214727), anti 5-hmC (Abcam, cat. no. ab214728), anti 5-fC (Diagenode, cat. no. C15310200), and anti 5-caC (Diagenode, cat. no. C15410204-100). Genomic DNA (20 μg) was diluted in 450 μl of 1× IP buffer (10 mM Na_3_PO_4_ pH 7.0, 140 mM NaCl, and 0.05% Triton X-100). A total of seven pulses of 90% intensity for 15 s for each sample at 4 °C were delivered with a UP50H ultrasonic processor (Hielscher Ultrasonics GmbH, Teltow, Germany). The size of the sonicated DNA was verified on a 2% agarose gel with an average size of 400 bp and a range of from 200 bp to 800 bp. DNA was aliquoted to three 1.5 ml DNA LoBind® Tubes (Eppendorf-5 Prime, Inc., Boulder, CO, USA) to avoid sample surface binding. Samples were heat denatured at 95 °C for 10 min and then immediately cooled on ice for 10 min. A 7 μg aliquot of antibody was added and samples were incubated on a rotating platform overnight at 4 °C. DIP was performed using 50 μl of Invitrogen Protein G or A Dynabeads and incubated for 2 h on a rotating platform at 4 °C. Samples were washed three times with 1× IP buffer using a magnetic rack and suspended in digestion buffer (50 mM Tris pH 8, 10 mM EDTA, 0.5% SDS) with 1 μl of 20 mg ml^–1^ proteinase K and incubated for 1 h at 50 °C on a rotating platform. Untreated sonicated genomic DNA was processed in parallel and considered as the input sample. Precipitated DNA was recovered using a MinElute PCR Purification Kit (Qiagen). Once the DNA was purified and quantified, we proceeded with quantitative PCR (qPCR).

### DIP-qPCR

qPCR of immunoprecipitated DNA was performed as follows: 10 ng of input DNA used as a normalization control or immunoprecipitated DNA, 5 μM of each primer, and a SensiFAST HRM Kit (Bioline Reagents Ltd., London, UK) were mixed according to the manufacturer’s instructions and amplified using the Rotor-Gene Q (Qiagen, Dusseldorf, Germany) according to the program 95 °C for 3 min, 40 cycles at 95 °C for 5 s, and 60 °C for 30 s. A later melting step from 30 °C to 95 °C was carried out to test product specificity. The promoter region from each gene (500 bp upstream from the transcript starting site) was selected for amplification analysis. Four replicates for each transcript from a pool of individuals were analysed using the LinReg software (Ruijter *et al.*, 2009) (primer sequences are indicated in Supplementary Table S1). The data for the analysis were obtained from the enrichment ratio according to the formula IP DNA/IN DNA (IP DNA, immunoprecipitated DNA; IN DNA, input DNA) determined from the qPCR results. An internal standard control with a known sequence was included in the experiment. The binary vector pCAMBIA-2301 and the primers F: ACGTAAGGGATGACGCACA and R: CGCGATCCAGACTGAATGCC were used to generate an amplicon of 389 bp. In the PCR, for each reaction, cytidine was replaced with the corresponding modified nucleoside: 5-methyl-2'-deoxycytidine, 5-hydroxy-2'-deoxycytidine, 5-formyl-2'-deoxycytidine, and 5-carboxy-2'-deoxycytidine (TriLink BioTechnologies Inc., San Diego, CA, USA). The reaction was as follows: 10 µl of Buffer 5X, 5 µl of dNTP (10 µM), 1 µl of Q5 High Fidelity DNA Polymerase (New England Biolabs. Inc., Beverly, MA, USA), 2 µl of primers F+R (10 µM each), and 2 µl of DNA (1 ng µl^–1^); ddH_2_O was added to the reaction to bring the final volume up to 50 µl. The program used was: 98 °C for 30 s and 35 cyles at 98 °C for 10 s, 55 °C for 30 s, and 72 °C for 30 s. A final extension step at 72 °C for 2 min was included. The IP DNA/IN DNA ratio for each cytosine modification is presented in Supplementary Fig. S1. Three replicates for each immunoprecipitation were analysed using the LinReg software (Ruijter *et al.*, 2009)

### Statistical analysis

For the data analysis, Kruskal–Wallis test was applied for the comparison of global quantification. Euclidean distance was used for the clustering analysis from immunoprecipitation data. Finally, a *t*-test was employed for image analysis comparison between both X chromosomes in female and male plants of *S. latifolia* for 5hmC and 5fC signals. The data for this analysis were the mean values of the antibody signal/DAPI signal ratio. For all the statistical tests, a minimum of three replicates were used. Statistical analysis was done according to RStudio Team (2022).

### Slide preparation and immunostaining

Slide preparations were carried out according to Houben *et al.* (2003) with modifications. Immunostaining was performed using the following procedure: slides were fixed in 4% formaldehyde in phosphate-buffered saline (PBS) for 10 min and subsequently washed three times in PBS. Samples were denatured in 2 M HCl for 15 min at room temperature and washed three times in PBS. Blocking was performed in a 3% blocking buffer [3% (w/v) BSA, 0.1% Tween-20 in 1× PBS] at 37 °C for 60 min and followed by incubation at 4 °C overnight with primary antibody diluted in 1% blocking solution [1% (w/v) BSA, 0.1% Tween-20 in 1× PBS]. The primary antibodies used in this study included rabbit anti-5mC (1:1000, Abcam, cat. no. ab214727), mouse anti-5mC (1:1000, Diagenode cat. no. C15200003), rabbit anti-5hmC (1:200, Abcam, cat. no. ab 214728), rabbit anti-5fC (1:200, Active Motif, cat. no. 61223), and rabbit anti-5caC (1:100, Diagenode, cat. no. C15410204-100). After several washes in 1× PBS, the slides were incubated with secondary antibody diluted in 1% blocking solution [1% (w/v) BSA, 0.1% Tween-20 in 1 × PBS] at room temperature for 1 h. The secondary antibodies included fluorescein isothiocyanate (FITC) AffiniPure Fab fragment donkey anti-mouse IgG (H+L) (1:400, JacksonImmunoResearch, cat. no. AB_2340797), and Cy™3 AffiniPure Fab fragment goat anti-rabbit IgG (H+L) (1:400, JacksonImmunoResearch, cat. no. AB_2313593). In double immunostaining experiments (5mC+5hmC and 5mC+5fC), the two primary or secondary antibodies were incubated together. After secondary antibody incubation, slides were washed three times for 10 min in PBS, dehydrated in an ethanol series, and mounted in Vectashield containing DAPI (Vector Laboratories, cat. no. GZ-93952-27). A negative control was included for all antibodies (Supplementary Fig. S2).

### Image acquisition and processing

Fluorescence images were taken in super resolution mode on a Zeiss Axio Observer.7 inverted microscope with laser scanning unit LSM 880 and Airyscan detector. The Plan-Apochromat ×63/1.4 oil DIC M27 objective was used for all imaging. Fluorescence signals were recorded using appropriate laser excitations: for DAPI, 405 nm; and Cy3, 561nm. Images were taken using an Axiocam MRm monochromatic camera driven by ZEN Black software. Recorded Airyscan super-resolution images were analysed by Zen Blue software (version 3.0) and images were deconvoluted using the Airyscan Joint Deconvolution function, and 3D reconstructions were produced using Imaris software (version 9.3, Bitplane, Zurich, Switzerland). Image quantification for DAPI and antibody signal was performed using Fiji (Schindelin *et al.*, 2012). The whole chromosome length and width from the maximum projection were used for the measurements and statistical analysis. The plugin colour profiler was used to split the RGB channels and calculate both the mean values for the global image analysis and the data for the graphical signal distribution in the sex chromosomes. The selected sex chromosome pairs in the figures came from the same metaphase in females. For males, not all the images came from the same metaphase, and all the figures used for the artwork can be found in Supplementary Fig. S3–S6 and in the Zenodo repository (https://doi.org/10.5281/zenodo.8386048).

### Quantification of 5mC and oxi-mCs in DNA.

The genomic content of C-5-modified nucleobases was determined by quantitative analysis of the corresponding deoxynucleosides after enzymatic hydrolysis of DNA performed using a modification of the method described by Starczak *et al.* (2021). Briefly, DNA samples were completely dried in a SpeedVac system. Next, the pellet was dissolved in 50 µl of MilliQ-grade deionized water and mixed with 50 µl of NP1 buffer (200 mM ammonium acetate, 0.2 mM ZnCl_2_; pH 4.6). Nuclease P1 (100 U, New England Biolabs) and tetrahydrouridine (10 mg ml^–1^) were added to the mixture and incubated at 37 °C overnight. Subsequently, 13 µl of 10% (v/v) NH_4_OH and 12 U of shrimp alkaline phosphatase (rSAP, New England Biolabs) were added to each sample following 2 h incubation at 37 °C. All hydrolysates were ultrafiltered prior to injection and concentrated in a SpeedVac to a final volume of 10 µl.

DNA hydrolysates were spiked with a solution of internal standard in a 4:1 volumetric ratio, to a concentration of 50 fmol µl^–1^ [D_3_]-5-(hydroxymethyl)-2'-deoxycytidine (5hmdC), [^13^C_10_, ^15^N_2_]-5-formyl-2'-deoxycytidine (5fdC), and [^13^C_10_, ^15^N_2_]-5-carboxy-2'-deoxycytidine (5-cadC), and analysed using isotope dilution automated online 2D-UPLC-MS/MS.

Chromatographic separation was performed in the 2D-UPLC system with a photodiode array detector for the first dimension chromatography (used for the quantification of canonical deoxynucleosides) and a tandem quadrupole mass spectrometer (Xevo TQ-XS, Waters), using a Waters Cortecs T3 column (150 mm×3 mm, 1.6 µm) with a pre-column for the first dimension, a Waters X-select C18 CSH column (100 mm×2.1 mm, 1.7 µm) for the second dimension, and a Waters X-select C18 CSH column (20 mm×3 mm, 3.5 µm) as a trap/transfer column. For the first dimension, a flow rate of 0.5 ml min^–1^, an injection volume of 2 µl, and gradient elution for 10 min was applied, using a mobile phase of 0.05% acetate (A) and acetonitrile (B) (0.7–5% B for 5 min, followed by column washing with 30% acetonitrile and re-equilibration with 99.3% A for 3.6 min). For the second dimension, the flow rate was 0.35 ml min^–1^ in a gradient elution for 10 min using a mobile phase of 0.01% acetate (A) and methanol (B) (1–50% B for 4 min, isocratic flow of 50% B for 1.5 min, and re-equilibration with 99% A up to the next injection).

All the samples were analysed in 3–5 technical replicates, and the technical mean was used for further calculation. Transition patterns for all the analysed compounds along with specific detector settings are given in Supplementary Table S2. The quantities of canonical deoxynucleosides were determined by UV detection at 260 nm for 2'-deoxythymidine (dT), and at 280 nm for 2'-deoxyguanosine (dG), and 5-methyl-2'-deoxycytidine. The total deoxynucleoside amount (dN), calculated as double the sum of dT and dG, was used as a reference for quantitative expression of modified deoxynucleosides.

## Results

### Global levels of cytosine modifications showed variation, but sex was not the variable

**Fig. 1.**
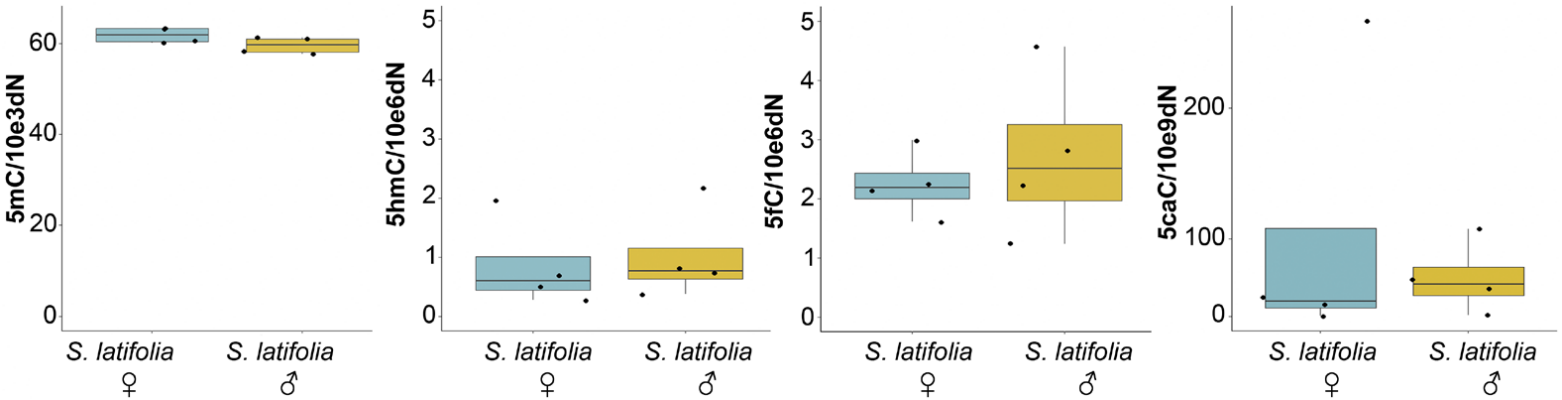
Boxplot graphical representation of 5mC, 5hmC, 5fC, and 5caC global content in *S. latifolia* female and male leaves. The box represents the interquartile range (IQR) defined as the range between Q3 and Q1. The line inside the box represents the median. The whiskers represent the most extreme data within the distribution. Individual data beyond the whiskers are considered as outliers. *n*=4. Black dots represent individual data points. Kruskal–Wallis rank sum test (*P*>0.05).

The first experimental approach was global quantification analysis for all the cytosine modifications in the genomic DNA of *S. latifolia* male and female plants. The most abundant cytosine modification was 5mC, with a median value of 59.61 modifications per kilobase for males and 61.90 modifications per kilobase for females Fig. 1. For the oxidative forms 5hmC and 5fC, the levels were three orders of magnitude less, and 5caC was six orders of magnitude lower than 5mC. The statistical analysis did not show significant differences between male and female plants for any of the modifications under analysis.

### 5mC, 5fC, and 5caC are preferentially localized in DAPI-dense regions in contrast to 5hmC

**Fig. 2.**
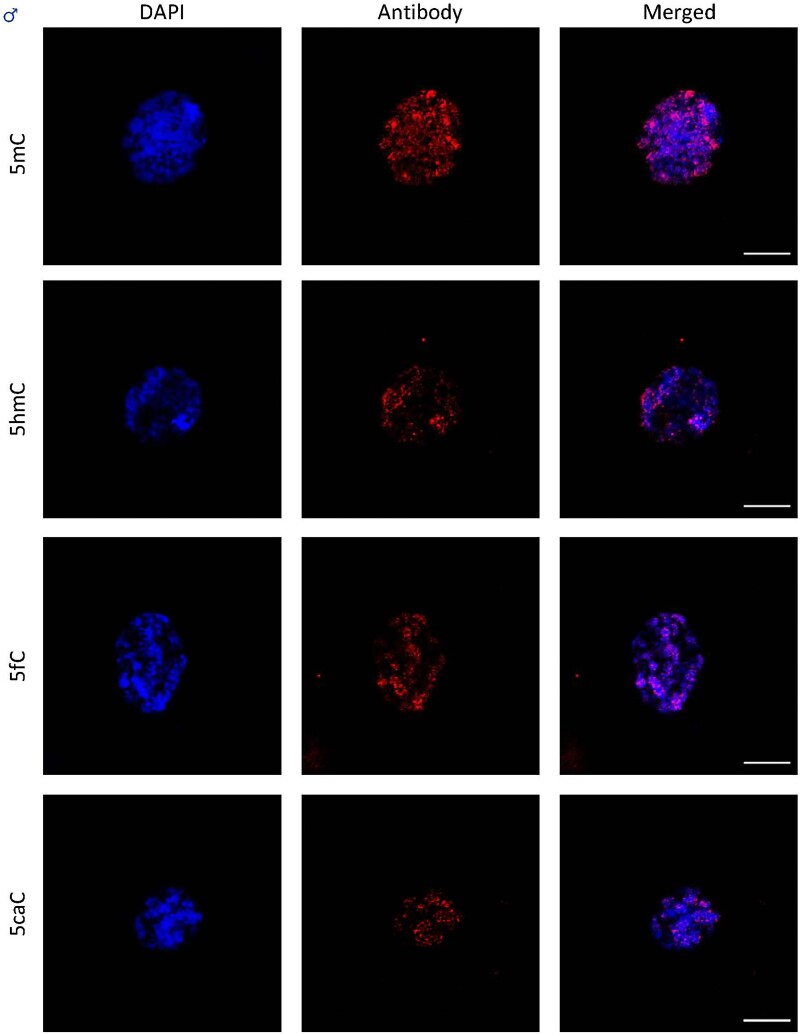
Immunodetection of oxi-mCs in *S. latifolia* males. The nuclei (DAPI) are shown in blue and the signals of oxi-mCs are represented in red (ANTIBODY). The bar represents 10 µm.

**Fig. 3.**
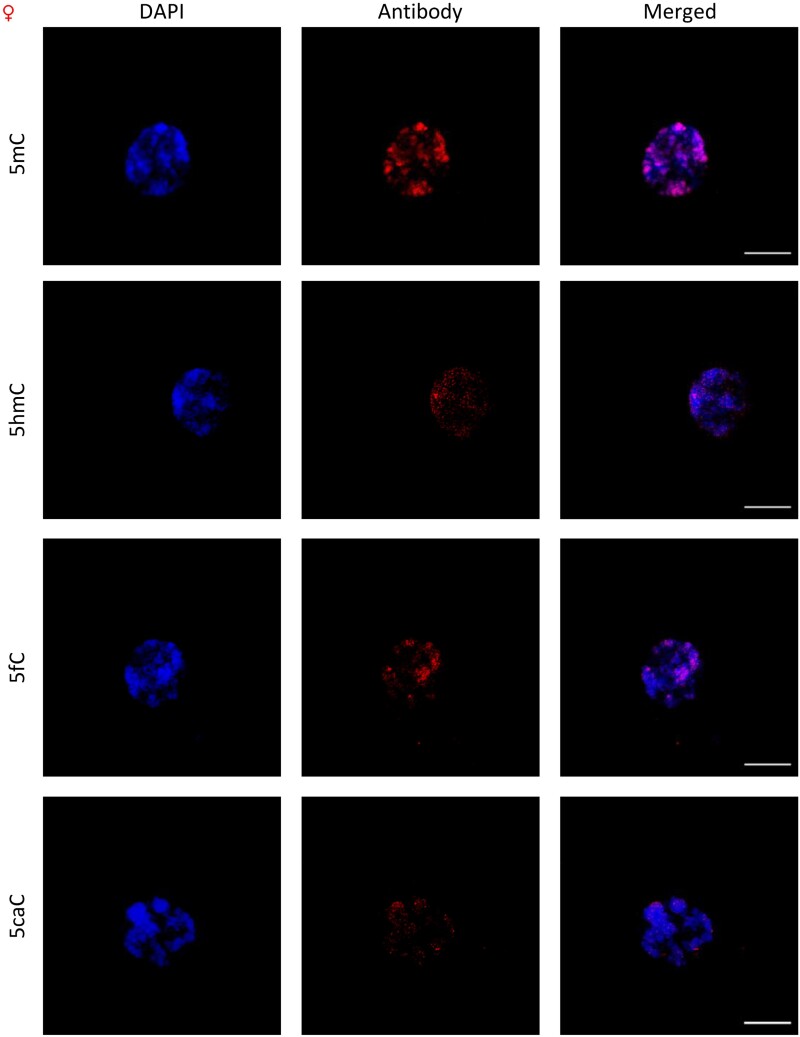
Immunodetection of oxi-mCs in *S. latifolia* females. The nuclei (DAPI) are shown in blue and signals of oxi-mCs are represented in red (ANTIBODY). The bar represents 10 µm.

The fluorescence signal pattern in the different nuclei (Figs 2, 3) clearly demonstrates co-localization of 5mC, 5fC, and 5caC with DAPI-dense territories in both sexes, which are usually considered as heterochromatin-related regions. The aforemetioned 5hmC signal was distributed throughout the nuclei on the other hand, and appeared to be present in small uniform size speckles in both sexes. The same signal pattern was observed in metaphase chromosomes in both sexes (Supplementary Figs S3–S6).

### A differential distribution on one of the X chromosomes in females depends on 5mC, 5hmC, and 5fC

**Fig. 4.**
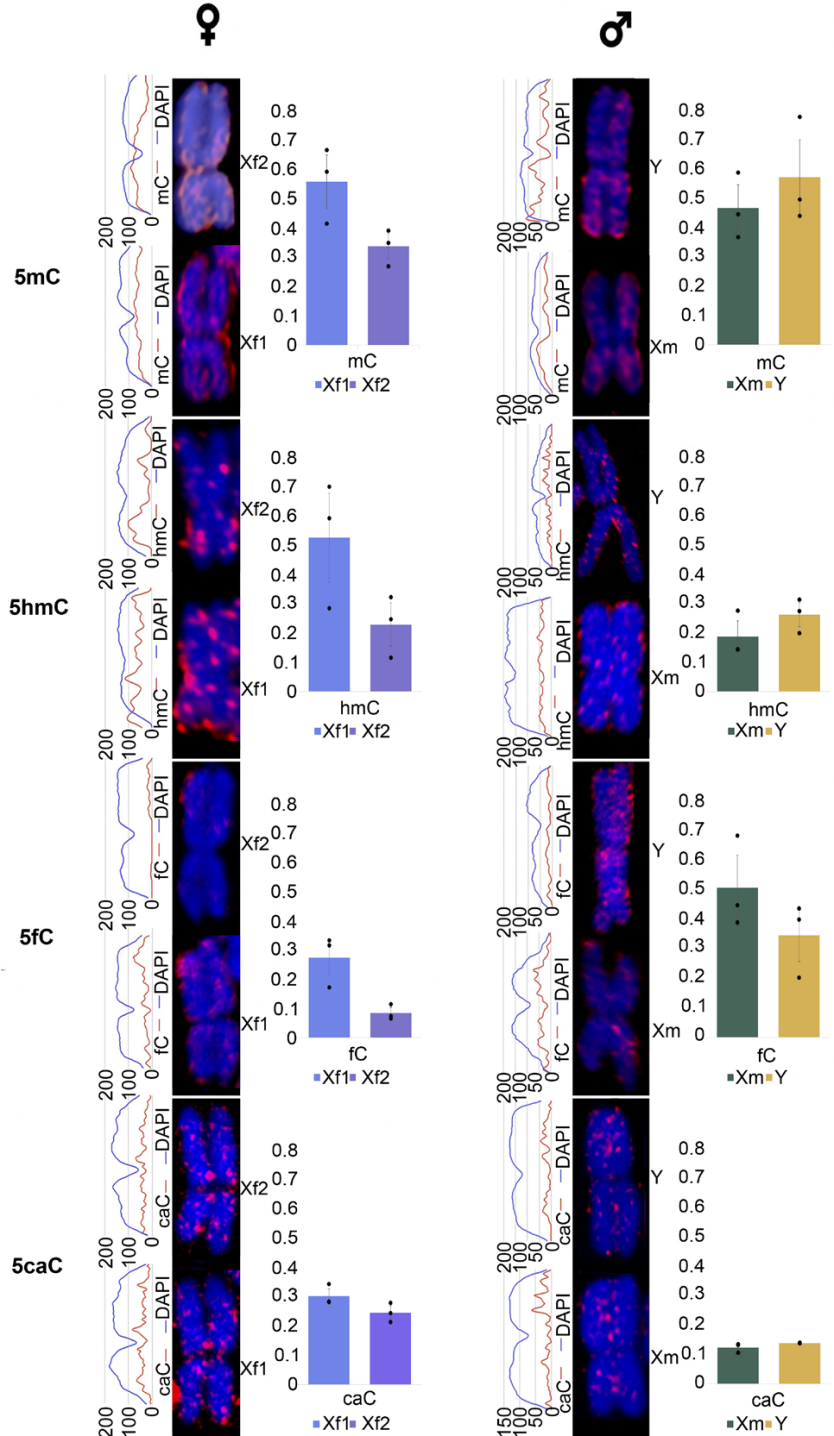
Magnification detail of 5mC, 5hmC, 5fC, and 5caC immunolocalization in metaphasic *Silene latifolia* female X chromosomes and male X and Y chromosomes stained with DAPI. A graph for the associated fluorescence along each chromosome length for antibody versus DAPI signals is shown on the left of the chromosome images. The relative antibody/DAPI mean fluorescence ratio for both X sex chromosomes in females and X and Y chromosomes in males is shown on the right of the chromosome images. For all the bar plots, *n*=3. Black dots represent individual data points. Asterisks represent *t*-test *P*-value (*P<0.05).

**Fig. 5.**
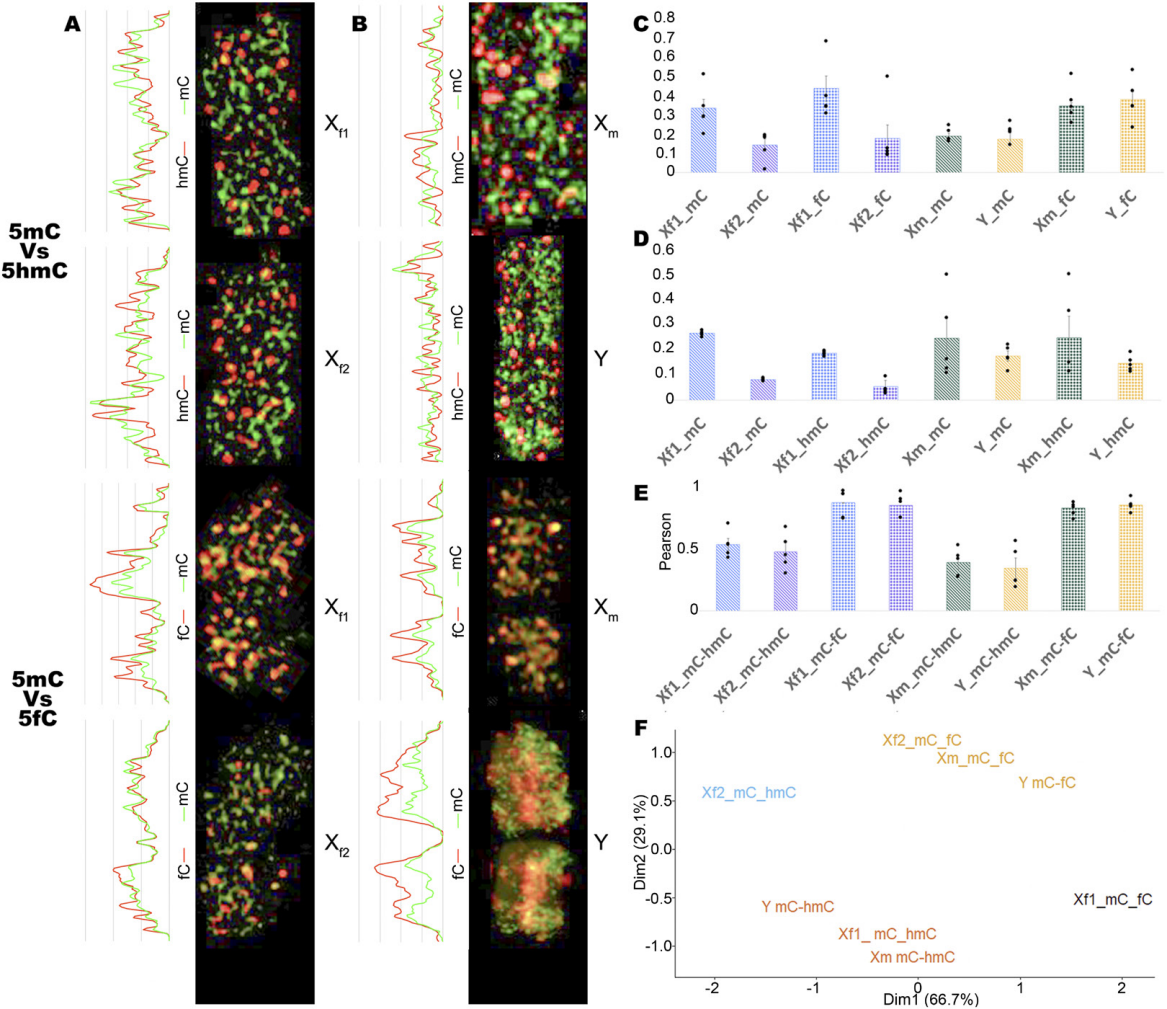
*Silene latifolia* sex chromosome landscape of co-localization of 5mC with 5hmC and with 5fC. (A) Magnification of 5mC–5hmC and 5mC–5fC co-localization output in metaphasic *Silene latifolia* female X chromosomes. A graph for the associated fluorescence along each chromosome length for each antibody combination is shown on the left of the chromosome images. (B) Normalized antibody/DAPI mean fluorescence ratio for both X sex chromosomes for the antibody combination 5mC–5hmC and 5mC–5fC, and Pearson mean correlation for each combination correlation. For all the bar plots, *n*=4. Black dots represent individual data points. Asterisks represent *t*-test *P*-values (*P<0.05, **P<0.01).

We set a nomenclature to distinguish among X sex chromosomes: X_f1_, the X chromosome from females 1, X_f2_, X chromosome from females 2; and X_m_, X chromosome from males. In all situations, X_f1_ was the chromosome with the highest 5mC signal. For all the antibodies except that against 5caC, chromosome X_f2_ showed a lower fluorescence signal than its counterpart X_f1_ (Fig. 4). X_f2_ has arms displaying a higher intensity of 5mC and 5hmC. For 5fC, however, the long arm has the higher intensity signal. Based on the total antibody/DAPI ratio for X chromosomes, differences (*t*-test; *P*-value<0.05) were found between both chromosomes for 5mC, 5hmC, and 5fC. Differences were not observed between X_m_ and Y chromosomes, or between X_f1_ and X_f2_ compared with X_m_. However, X_m_ displayed the same differential staining along the chromosome as X_f2_, according to the associated fluorescence graph. In this chromosome, 5mC and 5hmC signal was mostly localized in the short arm while 5fC had an opposite trend. An association for both X chromosomes in females with 5hmC or 5fC compared with 5mC revealed in all cases that X_f1_ was the chromosome with the higher signal (Fig. 5A–D). According to the observations made on the associated fluorescence graphs (Fig. 5A, B), both antibody signals had a similar pattern for 5mC–5fC. However, for 5mC–5hmC the pattern was different. Co-localization analysis detected differences between 5hmC and 5fC co-localization with 5mC (Fig. 5E). This difference is always present regardless of the chromosome and the sex, with a higher co-localization for 5mC and 5fC. A clustering analysis groups each female X chromosome differently with the two sex chromosomes in males (Fig. 5F). This is based on 5hmC or 5fC, but both oxi-mCs display a higher signal on chromosome X_f1_, which could indicate a common association of chromosome X_f2_ based on 5hmC together with chromosomes X_m_ and Y. On the other hand, the clustering of chromosomes X_f1_, X_m_, and Y may indicate a common connection dependent on the combination of 5fC and 5mC.

### Selected sex-linked genes and bias-represented TEs are clustered according to oxi-mCs

**Fig. 6.**
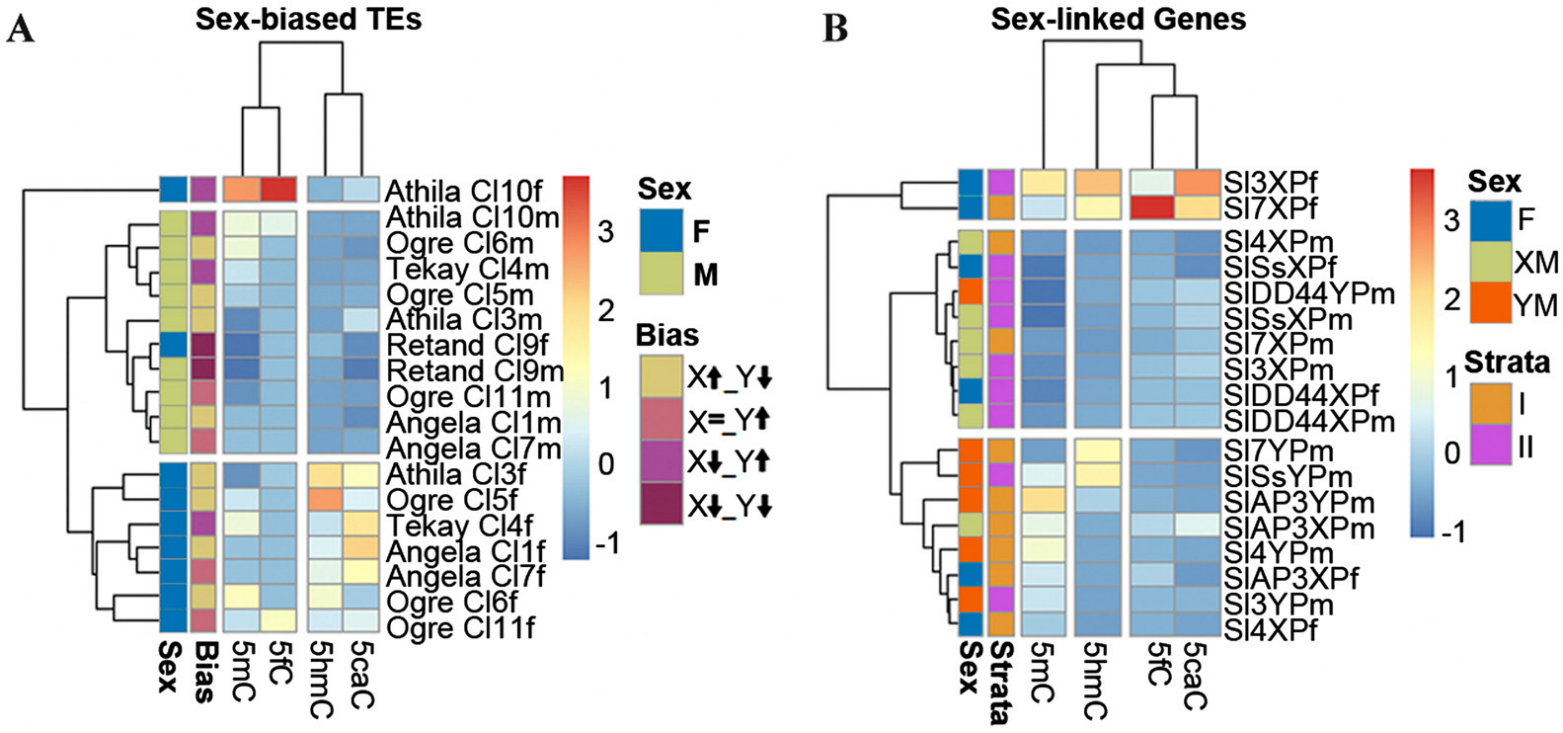
Distance matrix from the enrichment of oxi-mCs performed on male and female *S. latifolia* plants represented by a heatmap and the associated dendrogram. (A) Data from immunoprecipitation for sex-biased TE clusters. Additional annotation includes sex, TE family, and bias representation. Bias labelling code: X↑, over-represented in X chromosome; X↓, under-represented in X chromosome; X=; no changes in X chromosome; Y↑, over-represented in Y chromosome; Y↓, under-represented in Y chromosome. For each TE cluster, a combination of one X and one Y is applied. (B) Data from immunoprecipitation include X and Y alleles from selected sex-linked genes. Additional annotation for sex and strata is included. Sex labelling code: F, both X alleles in female plants; XM, X allele in male plants; YM, Y allele in male plants.

The promoter regions for the X and Y alleles from the genes *SlAP3*, *SlDD44*, *Sl3*, *Sl4*, *Sl7*, and *SlS* are already known to be involved in epigenetic regulation and were investigated in this work (Rodríguez Lorenzo *et al.*, 2018, 2020). The selection of the sex-biased TEs was according to Cermak *et al.* (2008), Kubat *et al.* (2008), Kralova *et al.* (2014), and Puterova *et al.* (2018), and the selected TEs were: *Tekay Cl.4*, *Angela Cl.7* and *Cl.1*, *Retand Cl.9*, *Athila Cl.10* and *Cl.3*, and *Ogre Cl.5*, *Cl.6*, and *Cl.11*. For this analysis, the enrichment raw data of the 5mC antibody from Rodríguez Lorenzo *et al.* (2018) were included. TEs show common clustering based on sex, indicating a different regulation in males and females (Fig. 6A). The grouping suggests that females are linked to 5hmC and 5caC. *Athila Cl.10* tested in females has its own branch and it is connected to 5mC and 5fC. The different alleles in the dendrogram cluster in such a way as to distinguish between X and Y chromosome in males is shown in Fig. 6B. This clustering is also maintained for strata. In the differential clustering between X and Y alleles in males, the X alleles in females share the same position in the branch. This finding, independently of the parent, may indicate a connection among all X alleles based on the oxi-mCs and a different behaviour for the Y alleles. In addition, this connection is extended in strata I (older) and II (younger) of the Y chromosome. The results suggest that alleles from the Y chromosome belonging to stratum I could be mainly associated with 5mC. The alleles *Sl3X* and *Sl7X* tested in females clustered in an independent branch. These alleles show high levels for all the modifications; however, they are clustered to 5fC and 5caC.

The clustering according to oxi-mCs reflects differences in both dendrograms, indicating a different relationship between genes and TEs. For TEs, 5mC clusters with 5fC, while 5hmC clusters with 5caC. For genes, 5fC and 5caC cluster together and 5mC and 5hmC remain single. This differential clustering may indicate a sex-dependent connection for TEs and a chromosome-dependent connection for genes.

## Discussion

Despite oxi-mCs being less represented compared with other cytosine modifications, we were able to detect 5caC in genomic DNA. A study in Norway spruce failed to detect 5caC using the same methodology (Yakovlev *et al.*, 2019). One possible explanation could be that the levels of 5mC in *S. latifolia* are 2–3 times higher, and the levels of oxi-mCs were 10 times higher. Because levels of oxi-mCs are connected to the levels of DNA methylation, the elevated levels of all 5mC precursors can be a consequence of active DNA demethylation. These results agree with the findings reported previously by Tang *et al.* (2014), showing similar levels of 5fC and 5caC in a number of plant species. Moreover, in the fungus *Coprinopsis cinerea*, a bias for 5fC production at the expense of other oxi-mCs has been shown (Zhang *et al.*, 2014). Comparing our global oxi-mCs levels in Fig. 1 with data from previous studies, it is important to point out that the presented results from tandem MS are even higher than levels reported in different mammalian tissues, where their biological function was directly described (Wu *et al.*, 2011; Gackowski *et al.*, 2015; Wu and Zhang, 2017). For *S. latifolia*, our main goal was not only to see the differences between the sexes, but also to show the signal distribution in interphase nuclei in Figs 2 and 3 and metaphase chromosomes, especially in the sex chromosomes represented in Fig. 4. Co-localization of 5mC with DAPI-dense foci was expected according to Tariq *et al.* (2003). These foci are considered to be heterochromatin linked with a dominant contribution in repetitive DNA (Fransz *et al.*, 2002). Despite 5fC and 5caC following the same trend, the distribution of 5hmC was different. A dispersed signal throughout the nuclei (Figs 2, 3) with a spotted signal pattern indicated their presence in euchromatic regions. Potentially, this difference could reflect a difference in the function of 5hmC which is in agreement with the same observations reported in mammalian embryonic stem cells (Szulwach *et al.*, 2011; Kubiura *et al.*, 2012).

Previous studies suggested the evolution of partial dosage compensation in *S. latifolia*, (Muyle *et al.*, 2012, 2018; Krasovec *et al.*, 2019; reviewed in Muyle *et al.*, 2022). Cytological experiments carried out in *S. latifolia* have proved that one X chromosome is enriched in histone repressive marks, 5mC, and is late replicating (Siroky *et al.*, 1998; Bačovský et al., 2019). The 5mC observations agree with previous studies and, additionally, we found that 5hmC and 5fC selectively mark the same X chromosome (Figs 4, 5). It is worth noting that a biased distribution of 5hmC has also been observed between X chromosomes in mice (Bogomazova *et al.*, 2014). On the other hand, in this work, the positively stained X chromosome was associated with the active status and an accumulating 5hmC signal has been described during the silent X chromosome reactivation. During our study, it was also observed that sister chromatids in metaphase occasionally stained differentially for oxi-mCs, and this was random and not specific for any chromosome (Supplementary Figs S3, S4). These observations are consistent with those from Kubiura *et al.* (2012) and Bogomazova *et al.* (2014). The authors explained this phenomenon as a possible process of cumulative conversion between oxi-mCs. Published work on plants links 5hmC with silent TEs (Wang *et al.*, 2015), and in mammals is connected to embryonic stem cells and is inversely correlated to cell differentiation (Deniz *et al.*, 2019). On the other hand, 5fC is associated with hypomethylation and gene expression, and with transposon activation in oocytes (Inoue *et al.*, 2012; Raiber *et al.*, 2012; Neri *et al.*, 2015). Our data suggest a common behaviour for 5mC and 5fC that could be interpreted as silencing if we look at the main function of methylation, and this is supported by transposon silencing in fungi (Ličytė *et al.*, 2022). Likewise, the low correlation between 5hmC and 5mC agrees with previous research published in rye (Kalinka *et al.*, 2023). This aspect might indicate that 5hmC could have a dual activation/repression role. However, this fact remains to be seen through global genomic analysis of chemically modified DNA to identify the cytosine context.

Working with *S. latifolia* offered the possibility to include in this work both sex-biased TEs and selected sex-linked genes. The epigenetic regulation of these genes in previous analyses involving DNA methylation and histone modifications detected differences between X and Y alleles and was one of the reasons for the inclusion (Rodríguez Lorenzo *et al.*, 2018, 2020). The second was the degree of degeneration that these genes have (Marais *et al.*, 2008), including mutations and substitutions. Bolstering their inclusion in this investigation, a specific gene regulation by 5hmC based on preventing spurious transcription has been reported in humans (Pfeifer, 2023; Wu *et al.*, 2023). Based on the degeneration of these genes, 5hmC might have a role in the fidelity of gene transcription and could be the cause of the difference found in *Sl7* and *SlS* promoters between X and Y alleles (Fig. 6). Additionally, the fact that several Y allele promoters and introns have increased in length compared with the X alleles due to TE accumulation offers an opportunity for analysis of differential regulation (Marais *et al.*, 2008; Blavet *et al.*, 2015). This fact nonetheless opens up the path to other options. In fungi, there are lineage-specific expansions with numerous TET/JBP genes, which are often associated with a unique class of TEs (Iyer *et al.*, 2014). A previous accumulation of repetitive DNA before gene degeneration (Kubat *et al.*, 2008), and a possible sex-specific transposon silencing (Puterova *et al.*, 2018) have been reported. Epigenetic silencing is the main mechanism to regulate sex-biased TEs in *S. latifolia* (Kubat *et al.*, 2014), and 5hmC has been linked to silent TEs and genes in rice (Wang *et al.*, 2015). In this work, we show sex-specific TE clustering based on enrichment of oxi-mCs (Fig. 6A). Given specific sex-biased TE clusters, which are linked to sex chromosome evolution, and the reality that no information is available for plant TET/JBP genes, all the possibilities should be taken into account. In fungi there is evidence for the association of TET/JBP genes with transposons (Iyer *et al.*, 2014) and, in mouse embryonic stem cells, different TETs bind different TE classes, showing 5hmC linked to LINE-1 elements (De La Rica *et al.*, 2016). The sex clustering (see Fig. 6B) is not as clear in genes as it is in TEs, but the clustering gathers the majority of X alleles from females and from males separate from the Y allele in males. It is crucial to remember that X chromosomes recombine opposite to the Y chromosome (Zluvova *et al.*, 2007). In the same way, the Y alleles from males clustered in stratum I linked to 5mC and different histone modifications, all of them repressive marks associated with genes in the Y chromosome (Rodríguez Lorenzo *et al.*, 2018, 2020). The clustering according to the oxi-mCs reflects differences in both dendrograms, indicating different association between genes and TEs (Fig. 6). For TEs, 5mC clusters with 5fC while 5hmC clusters with 5caC (Fig. 6A). This finding aligns with the strong correlation observed in the double immunohistochemistry, not only in the sex chromosomes but also in the autosomes (Supplementary Figs S5, S6).

The intricate interplay between epigenetic modifications and sex chromosome evolution remains a fascinating subject of investigation. Three key milestones mark this process: sex determination, dosage compensation, and Y chromosome degeneration. In *S. latifolia*, we can find evidence narrowing down a possible epigenetic regulation for all of them (Muyle *et al.*, 2018; Rodríguez Lorenzo *et al.*, 2018, 2020; Bačovský *et al.*, 2022; Akagi *et al.*, 2023, Preprint), and this study provides evidence of a connection for oxi-mCs in female sex chromosome evolution and Y chromosome degeneration, paving the way for further research to elucidate a hypothetical specific function in this ideal plant model for oxi-mC analysis.

## Supplementary data

The following supplementary data are available at *JXB* online.

Table S1. List of primers used in this work.

Fig. S1. Internal standards as DIP control.

Fig. S2. Immunohistochemistry-negative controls.

Figs S3 and S4. Raw images used for the artwork in this research for single immunohistochemistry assays.

Figs S5 and S6. Raw images used for the artwork in this research for double immunohistochemistry assays.

Table S3. Mass spectrometry standards.

erae178_suppl_Supplementary_Tables_S1-S2_Figures_S1-S6

## Data Availability

Raw image data are freely available in the Zenodo repository https://doi.org/10.5281/zenodo.8386048, (Hubinský *et al.*, 2024).

## Abbreviations

5caC: 5-carboxycytosine
5fC: 5-formylcytosine
5hmC: 5-hydroxymethylcytosine
5mC: 5-methylcytosine
oxi-mCs: enzymatic oxidation products of 5-methylcytosine
TET: ten–eleven translocation
X_f1_: female X chromosome number 1
X_f2_: female X chromosome number 2
X_m_: male X chromosome.
