# Supplementary material for: Non-canonical bases differentially represented in the sex chromosomes of the dioecious plant Silene latifolia
Source: J Exp Bot. 2024 Apr 23;75(13):3849–61. doi: 10.1093/jxb/erae178 (PMC11233409; doi:10.1093/jxb/erae178)
Supplement: erae178_suppl_Supplementary_Tables_S1-S2_Figures_S1-S6 [file erae178_suppl_supplementary_tables_s1-s2_figures_s1-s6.pdf]

Supplementary Table S1: List of primers used in this work

Gene-TE	Allele	Genbank Acc. CDS	Sequence 5'-3' Forward Oligo	Sequence 5'-3' Reverse Oligo	Amplicon lenght
SlDD44	*S. latifolia X*	*AJ631230*	AACAAGTCAATTCGTGTCATTTCG	GTGATCCGAATAGTGTGACTCGA	150
SlDD44	*S. latifolia Y*	*AJ631231*	TGTGGGTTAATAGATGGGGAAAGG	ACTCACATTTTCAAATCTTGTTCCT	157
Sl4	*S. latifolia Y*	*AJ310659*	TATTATCATCAATCTCAATACTCAGATCTGTTC	GATGATCTAGTTAGATTATAATCTGGGTCATTG	183
Sl7	*S. latifolia Y*	*EU561060*	CGATATTTACTAAAACGTGTCGATATAAATCAG	GTGTTATTCTTTCCTTGCTTTGTTTTACTTCTA	100
SlAP3	*S. latifolia X*	*AB519802*	AAAGAGGCTCATTGTCATACTACA	TGGAAAGTAGTAAGTGGATGTTAACT	164
SlAP3	*S. latifolia Y*	*AB519801*	ACCCGAAATAGACCCGAAACC	TTTGAGATGATTAGACATTACGTTAACT	101
SlSs	*S. latifolia X*	*AY705437*	AAAGGGCATTAACCAAAATCCG	ACAAGTTTGATTATTTTGAATTTCGGT	101
SlSs	*S. latifolia Y*	*AY705438*	AGGGTAAAGACACAGTTTCTTCCT	TCCATGTCTTACAAACGCAATCA	119
Sl3	*S. latifolia X*	*AJ631223*	GCTAAACCATTGGAGATACCCATG	ATAAGTTCATTTTAAGTTGTGGGCCA	100
Sl4	*S. latifolia X*	*AJ310660*	ATTGACTGAGAAGTGAGAAAGTACTCGTAAGTC	ATTGAAGAATTTACAGTGAAAAGAAGAGAAGAC	122
Sl7	*S. latifolia X*	*EU561058*	GGCATAATATCTTTTGTGAGTTTATATATGTCG	CCGGTAGAGTTACTTTAATGGAGAATATTTATT	306
Sl3	*S. latifolia Y*	*AJ631224*	TAATCTCTACTCACCTTAATCAAACCG	ACTCGGCCGATTACCCCAG	91
AngelaCL1			TGCGATCACAACTGTTGATCA	TTTACCCAGGGCCAATGCAT	700
AngelaCL7			TGCACCCAAGACGATCTGAG	GAGCTGGTGTCCTCCACAAA	796
AthilaCL10			GACCAAGACGCAACTCCAGA	TCAAACACATGAGGCGGGAA	839
AthilaCL3			TACCGGAGTCTCCTTGCTCA	GCACTAGGGTGTCATGGGTT	905
OgreCL11			TTCCCCAATGCTTGTAGGAG	ATCGACTCGAGGTTCTTTCG	369
OgreCL5			ACAGCCAGAACTCACCCTTG	GGAGTCGCCACCAATTTTTA	769
OgreCL6			ACCGGGTTCAAATACCCATT	CCCGTTCGAATTCCACTTTA	638
RetandCL9			GGCATATCCGCGTACTCACA	TTCGGGGTCACTTTATGGGC	826
TekayCL4			GTTCCTTGCGTCGAGGGTAA	ACATCCCAGCTAGTCCCAGT	785

Supplementary Table S2. Transition patterns, specific detector settings and sources of standards for analyzed deoxynucleosides.

**compound name**	**ionization mode**	**nominal molecular mass (Da)**	**pseudomolecular ion formulation**	**nominal parent ion (Da)**	**nominal daughter ion (Da)**	**ESI capillary (kV)**	**ESI cone (V)**	**collision energy (eV)**	**standard source**
5-(hydroxymethyl)-2'-deoxycytidine	positive	257	[M+H]+	258	124	1.2	15	10	Berry & Associates
[D_3]-5-(hydroxymethyl)-2'-deoxycytidine	positive	260	[(M+3)+H]+	261	127	1.2	15	10	Toronto Research Chemicals
5-formyl-2'-deoxycytidine	negative	255	[M-H]-	254	121	3.5	28	18	Berry & Associates
[$^{13}C_{10}$, $^{15}N_2$]-5-formyl-2'-deoxycytidine	negative	267	[(M+12)-H]-	266	128	3.5	28	18	own synthesis
5-carboxy-2'-deoxycytidine	negative	271	[M-H]-	270	110	3.5	20	20	Berry & Associates
[$^{13}C_{10}$, $^{15}N_2$]-5-carboxy-2'-deoxycytidine	negative	283	[(M+12)-H]-	282	116	3.5	20	20	own synthesis

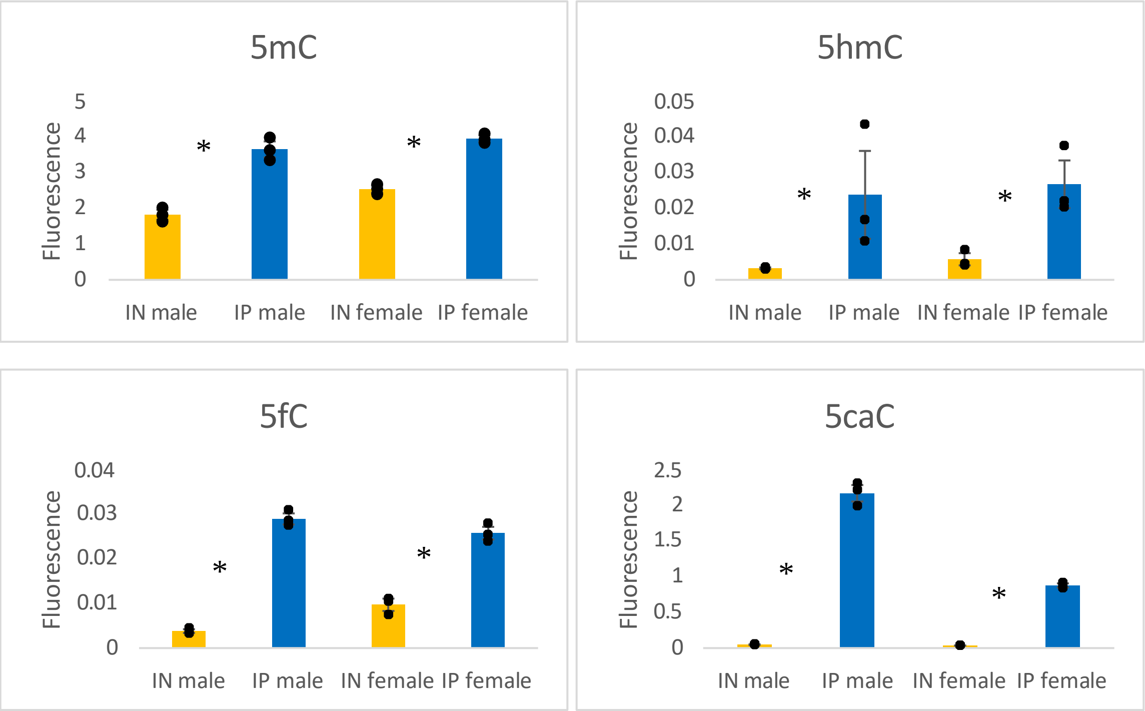

Supplementary Figure S1: qRT-PCR results for each antibody after immunoprecipitation in male and female plants. IN DNA Vs IP DNA (IN DNA: Input DNA, IP DNA: Immunoprecipitated DNA) The binary vector pCAMBIA-2301 was used to generate an amplicon of 389bp replacing the cytidine in the PCR reaction for the appropriate modified nucleoside (5-Methyl-2'-deoxycytidine, 5-Hydroxy-2'-deoxycytidine, 5-Formyl-2'-deoxycytidine and 5-Carboxy-2'-deoxycytidine) for each immunoprecipitation. n = 3. Asterisks represent statistical differences (* = p < 0.05) according to Kruskal-Wallis rank sum test (p>0.05)

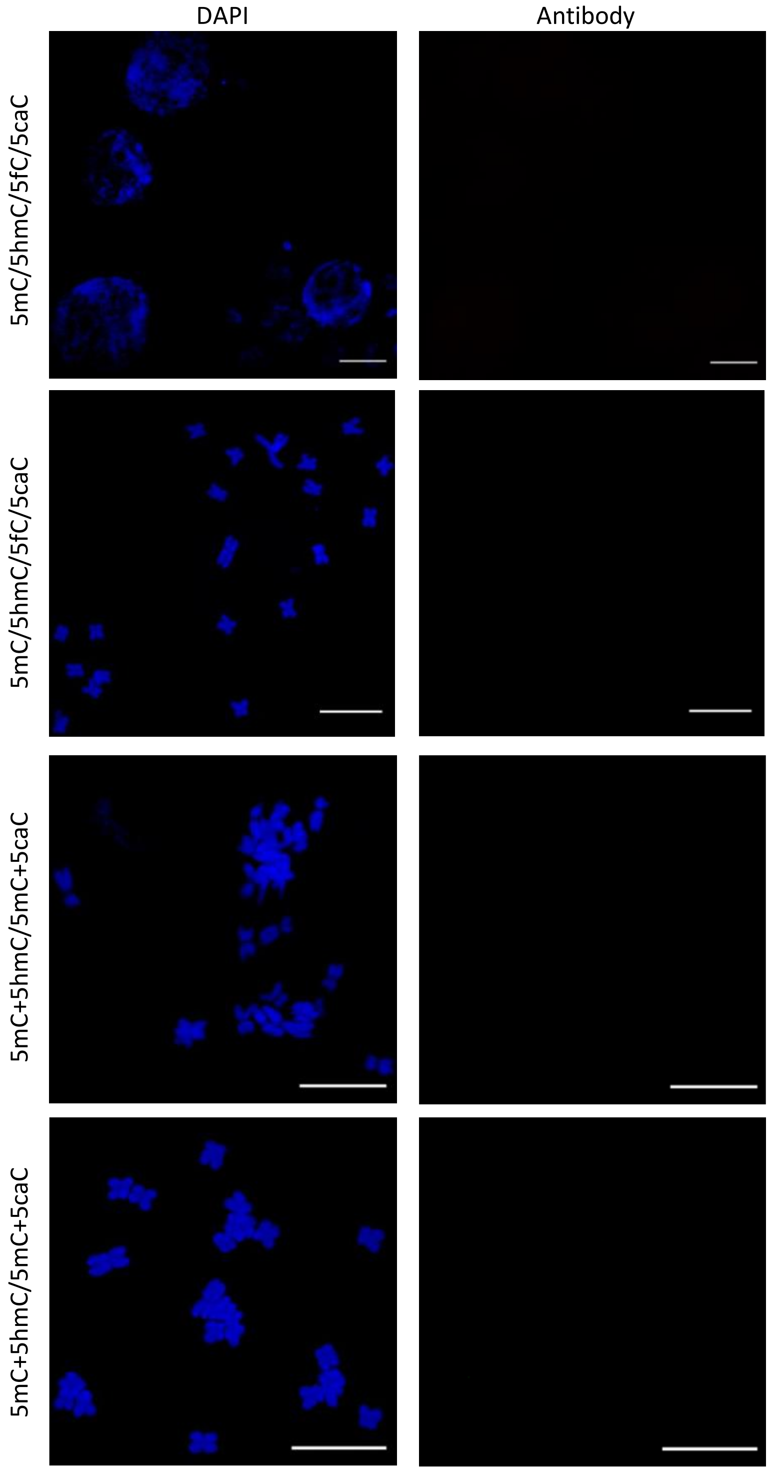

Supplementary Figure S2. Negative control of oxi-mCs immunofluorescence. “DAPI” staining of nuclei and chromosome in blue and “Antibody” - with the fluorescence generated by unspecific binding of the secondary antibodies. Microscope magnification is 63x. Bars are 10µ

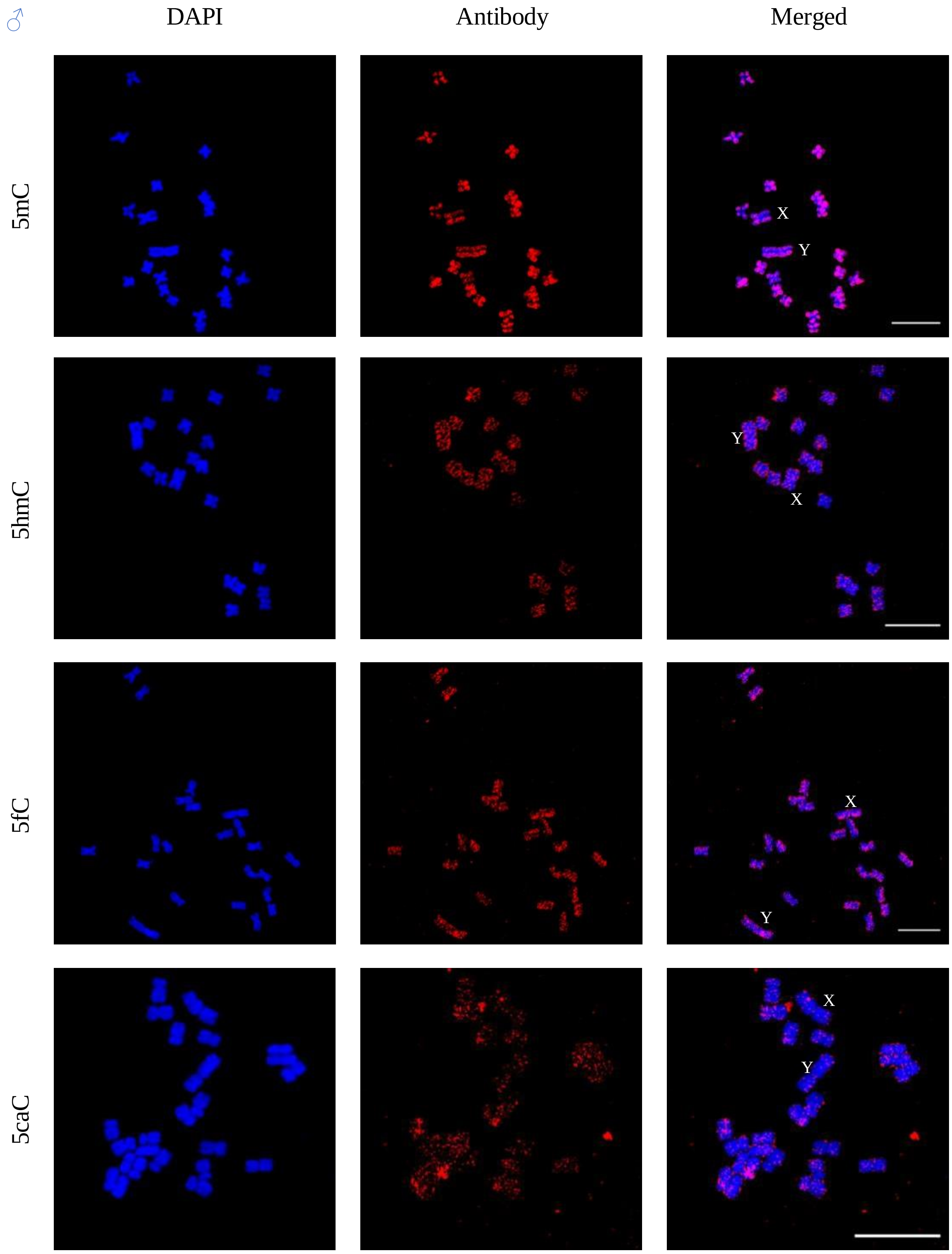

Supplementary Figure S3. Immunodetection of oxi-mCs in metaphasic chromosomes of *S. latifolia* male. DAPI is represented in blue, Antibody shows immunofluorescence signal and Merge represents signal overlay. The bar represents 10μm.

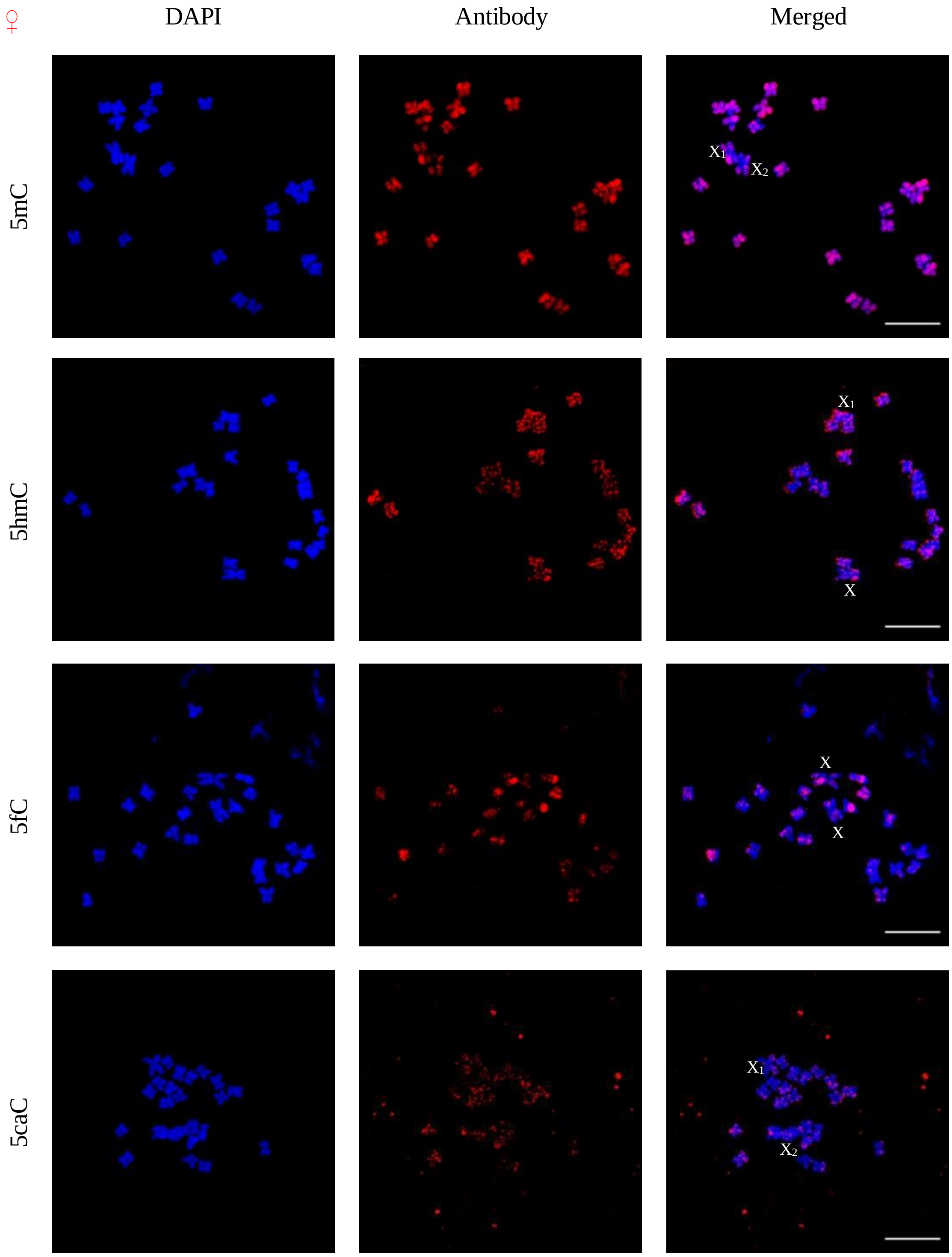

Supplementary Figure S4. Immunodetection of oxi-mCs in metaphasic chromosomes of *S. latifolia* female. DAPI is represented in blue, Antibody shows immunofluorescence signal and Merge represents signal overlay. The bar represents 10µm.

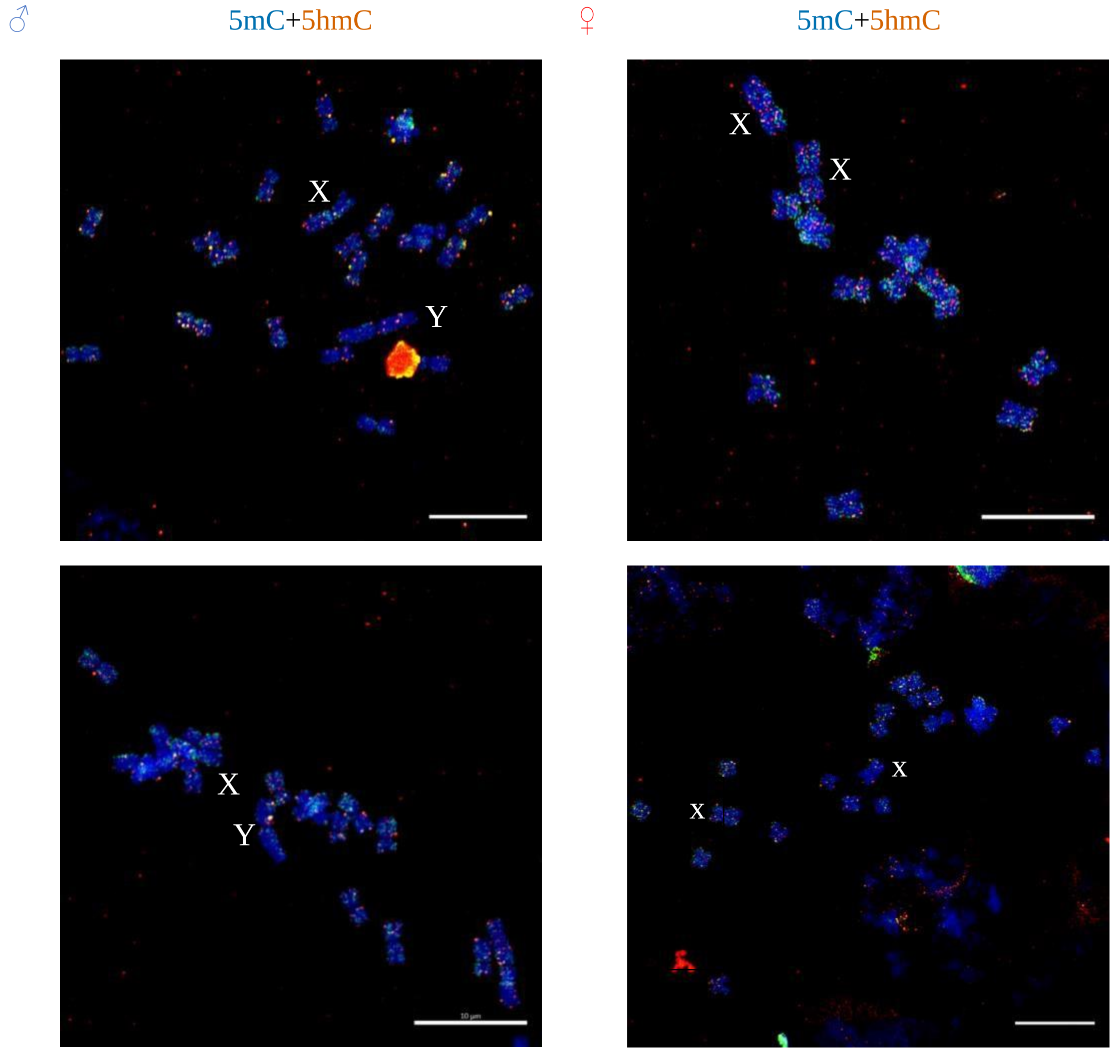

Supplementary Figure S5. Immunodetection of 5mC and 5hmC in *S. latifolia* male and female metaphasic chromosomes. DAPI is represented in blue, 5mC is represented in green and 5hmC is represented in red. The bar represents 10µm.

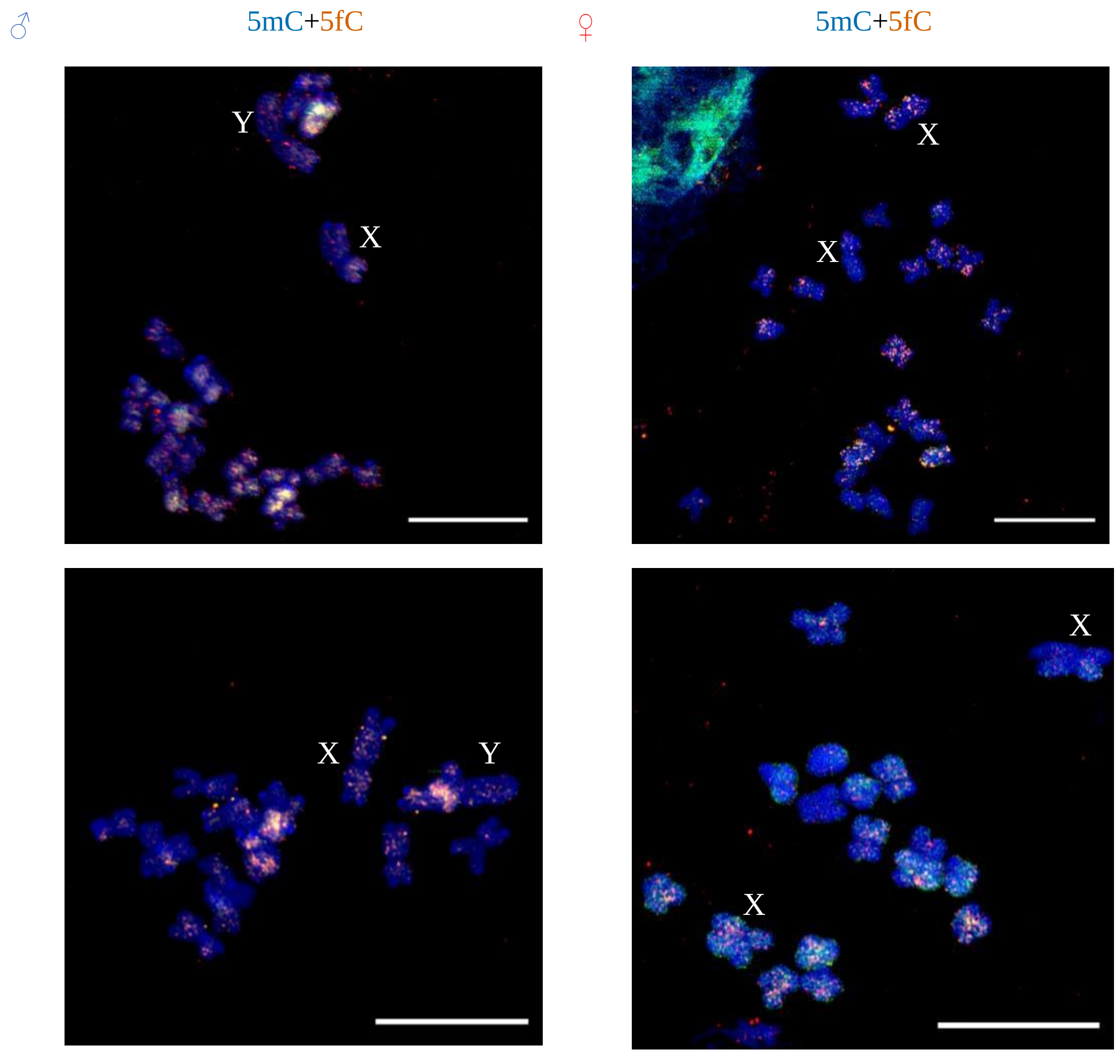

Supplementary Figure S6. Immunodetection of 5mC and 5fC in *S. latifolia* male and female metaphasic chromosomes. DAPI is represented in blue, 5mC is represented in green and 5fC is represented in red. The bar represents 10µm.
